# Epidermoid cyst of the clitoris

**DOI:** 10.11604/pamj.2021.38.59.27476

**Published:** 2021-01-19

**Authors:** Ahmed Ibrahimi, Adil Kallat

**Affiliations:** 1 Department of Urology A, Ibn Sina University Hospital, Faculty of Medicine and Pharmacy, Mohammed V University in Rabat, Rabat, Morocco,; 2 Provincial Hospital Center of Azilal, Bouznika, Morocco

**Keywords:** Epidermoid cyst, clitoral cyst, clitoromegaly, surgical excision, dysuria

## Image in medicine

An epidermoid cyst is a benign cyst resulting of implantation of superficial epidermal tissue into the dermis or subcutaneous tissue. They are commonly found on the scalp, face, neck, trunk and extremities; and rarely seen in the clitoral or vulvar region, most often associated with prior trauma or surgery to the area, particularly female genital mutilation. Epidermoid cyst of the clitoris is a very uncommon pathological entity, it can be spontaneous present at birth or develop later in life, or often secondary to surgery or trauma at the clitoral region. Herein, we report a case of 33-year-old multiparous woman, without past medical history of vulvar trauma or genital surgery, presented to the emergency department with a history of slowly growing clitoral mass over 11 months, which recently became responsible of sexual discomfort and difficulty in urination, without fever, vaginal discharge or other complaints. On perineal examination a painless midline cystic mass of 5 × 3 cm was noted; it extended from the clitoral region up to the labia minora; the mass was mobile, nontender, well-circumscribed and with a smooth surface (A, B). The labia minora and majora were normal, and both the urethral and vaginal openings were normal in position and shape (B). There was no associated bleeding or discharge. The remainder of the physical examination was otherwise unremarkable. Ultrasound testing revealed normal internal genital and urinary tract without communication between the cystic structure and the urethra. The patient underwent a complete surgical excision of clitoral cyst under spinal anesthesia, without compromising the clitoral neurovascular bundle or hood. Histopathological examination revealed an epidermoid cyst lined with stratified squamous epithelium and filled with keratinous material. The final pathologic diagnosis was of a spontaneous nontraumatic epidermoid cyst of the clitoris. The patient´s postoperative course was completely uneventful and she was discharged from the hospital on the 2^nd^ postoperative day without any complication. No recurrence of the cyst was noted 6 months later at a follow-up visit, and she had a normal cosmetic appearance of the external genitalia, with complete disappearance of genital and urinary complaints.

**Figure 1:**
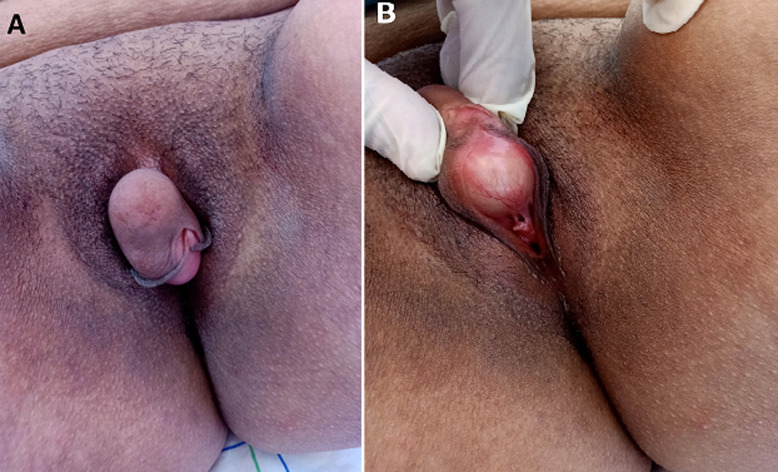
A) image of giant cyst of the clitoris occupying the vulvar inlet; B) clinical examination picture showing a large clitoral cyst with normal urethral and vaginal openings

